# Essential Paediatric Dermatology

**Published:** 1989-05

**Authors:** J. L. Burton


### Book Review

**Essential Paediatric Dermatology**, Julian Verbov. 1988, Clinical Press: Bristol. Pp. 182, Colour illustrations. Price £25.00.

This book is one of the first to be produced by a new Clifton-based publishing company, and it is absolutely first class.

The author, Julian Verbov, is a Consultant Dermatologist at the Royal Liverpool Children's Hospital and he already has a reputation as an excellent clinician who can also write well. The present book is aimed at general practitioners, paediatricians, and trainee dermatologists, though nurses and senior medical students would also find it helpful and interesting. It provides a concise and up-to-date illustrated account of the clinical features and treatment of the skin conditions which occur in babies and children in the U.K. and all but the very rarest diseases have been included.

The great strength of the book is the profusion of very high quality colour photographs, which not only beautifully illustrate the features described in the text, but have also been very well reproduced. One knows how difficult it can be to find the right patient, to take an excellent photograph of just the right area, and then to get the right colour tones and sharpness on the printed page, and the fact that all of the numerous pictures are so appropriate and so beautifully reproduced reflects great credit on the author and publisher.

The author's great clinical experience is reflected in the text, which although brief, gives a very readable and adequate summary of each condition. This histopathology is generally not mentioned however, and the treatment, though correct, is not discussed in any great detail. This brevity may well be regarded as a virtue by the hard-pressed G.P. however, and the book will be ideal for most examination candidates, though M.R.C.P. Paediatrics candidates might have welcomed a little more detail on a few conditions. Kawasaki's disease for example receives only the briefest mention as a variant of infantile polyarteritis nodosa, and the exanthemata such as measles and varicella are not included.

This book, which attractively printed in hard-back on high quality art paper, should be a great success and it augurs well for the future of this new publishing house.

J. L. Burton

